# Aflatoxin Contamination of Various Staple Foods from Angola and Mozambique

**DOI:** 10.3390/toxins16120516

**Published:** 2024-11-29

**Authors:** Cláudio Matusse, Zelda Lucamba, João Bila, Custódia Macuamule, Ana Sampaio, Sandra Afonso, Armando Venâncio, Paula Rodrigues

**Affiliations:** 1 CIMO, LA SusTEC, Instituto Politécnico de Bragança, Campus de Santa Apolónia, 5300-253 Bragança, Portugal; matusseclaudio8@gmail.com; 2 Department of Agriculture, College of Business and Entrepreneurship of Chibuto, UEM-Eduardo Mondlane University, Gaza 1200, Mozambique; 3 University of Trás-os-Montes and Alto Douro (UTAD), Quinta de Prados, 5000-801 Vila Real, Portugal; asampaio@utad.pt; 4 Instituto Superior Politécnico de Cuanza Sul, Rua 12 de Novembro, Sumbe, Cuanza Sul CP 82, Angola; zeldalucambalucamba@gmail.com (Z.L.); sandra.afonso3@gmail.com (S.A.); 5 Department of Crop Protection, Faculty of Agronomy and Forestry Engineering, UEM-Eduardo Mondlane University, Maputo 1102, Mozambique; jbilay@gmail.com; 6 Centre of Excellence in Agri-Food Systems and Nutrition (CE-AFSN), UEM-Eduardo Mondlane University, Maputo 1102, Mozambique; 7 Department of Animal Production and Food Technology, Faculty of Veterinary, UEM-Eduardo Mondlane University, Maputo 1102, Mozambique; custodiamacuamule@gmail.com; 8 Centre for the Research and Technology of Agro-Environmental and Biological Sciences (CITAB), University of Trás-os-Montes and Alto Douro (UTAD) Quinta de Prados, 5000-801 Vila Real, Portugal; 9 Laboratório Associado Instituto Para a Inovação, Capacitação e Sustentabilidade da Produção Agroalimentar (Inov4Agro), University of Trás-os-Montes and Alto Douro (UTAD), Quinta de Prados, 5000-801 Vila Real, Portugal; 10 Centro Nacional de Investigação Científica, Rua Avenida Ho Chi Minh, 201, Maianga, Luanda CP 34, Angola; 11 CEB—Centre of Biological Engineering, University of Minho, 4710-057 Braga, Portugal; avenan@deb.uminho.pt; 12 LABBELS—Associate Laboratory, 4800-058 Guimarães, Portugal

**Keywords:** toxicity, mycotoxins, food security, quality control, Africa

## Abstract

Aflatoxins constitute a significant risk in staple foods produced in African countries. This research aimed to analyze the total aflatoxin (AFT) contamination of various staple foods in Angola and Mozambique. A total of 233 samples of corn, peanuts, beans, rice, and cassava flour collected from farmers or local markets from the province of Cuanza Sul, Angola, and the provinces of Gaza and Inhambane, South Mozambique, were analyzed for the presence of AFT using the lateral flow strip method via AgraStrip^®^ Pro WATEX^®^ (Romer). The results showed that, from all matrices, the highest incidence and level of AFT were found in corn produced in Mozambique, with medians ranging from 6.5 to 66.5 µg/kg, with the samples showing values as high as 9200 µg/kg. Levels higher than the maximum admissible levels recommended by the Codex Alimentarius Commission for cereals and pulses (15 µg/kg) were observed in up to 90% of the corn samples, depending on the province. Corn produced in Angola showed lower amounts of AFT, with medians ranging from 1.2 to 7.7 µg/kg. Considering the maximum admissible levels for AFT recommended by the European Commission and the Codex Alimentarius Commission for cereals and pulses, the level of AFT contamination in staple food produced and consumed in the studied provinces is high and constitutes a public health risk for the population. Therefore, risk mitigation strategies are urgently needed.

## 1. Introduction

Aflatoxins (AFs) are considered the most harmful mycotoxins [1], and aflatoxin B1 (AFB1) in particular has been classified into Group 1 “carcinogenic to humans” by the International Agency for Research on Cancer [2]. Acute exposure to high doses can cause vomiting and abdominal pain, and, in extreme situations, it can be lethal, while chronic exposure at lower doses is associated with liver cancer [3]. Aflatoxins are among the most common mycotoxins in agriculturally important food crops worldwide. They thus constitute a major risk to human and animal health.

Aflatoxins contaminate many agricultural products, which are particularly susceptible to being infested by aflatoxigenic fungi [4]. Corn, peanuts, rice, sorghum, and wheat are each responsible for more than 10% of the global exposure to AFs [5]. Poor hygiene during transport and storage, high temperatures and relative humidity, and heavy rainfall are all conditions that favor fungal growth and potentiate AF production [6]. The populations in any region or country where these conditions are found are more susceptible to mycotoxin exposure. In general, these regions also face higher malnutrition and food insecurity problems, and there are few regulatory instruments that can protect the exposed and vulnerable populations.

African countries, particularly those belonging to the Southern African Development Community (SADC), are considered highly vulnerable to AF exposure. In such countries, corn and peanuts are base staple crops for most of the population and constitute the major source of AF intake for these populations. Despite this predicted high susceptibility, only a few countries in this region, as is the case for South Africa and Tanzania, have been subject to considerable research on AF incidence in their agricultural products, and the characterization of AF exposure in other countries in this region is scarce.

Angola and Mozambique are SADC countries with little or no knowledge of their staple foods’ AF incidence and contamination levels. Only a few reports are available from Mozambique, and, to our knowledge, none are available from Angola. In Mozambique, AF contamination has been reported for corn [7,8,9,10], peanuts [8,11,12], and cashew nuts [13].

The maximum tolerable limit (MTL) of AFs regulated by the European Union (MTL-EU) in peanuts and other oilseeds used as the only ingredient; products processed from peanuts; cereals; and products derived from cereals is very low: 4 µg/kg [14]. The AF MTL stipulated by the Food and Agriculture Organization (FAO) and Codex Alimentarius (MTL-Codex) for African countries is, on the other hand, less stringent: 10 µg/kg for peanuts, beans, cassava, and rice, and 15 µg/kg for corn [15]. In Mozambique, MTLs are established for peanuts only, at 10 µg/kg, and Angola has no established MTLs [15]. African countries have the sovereignty and right to apply the codex regulations for quality control and food safety (specifically mycotoxins). Still, exportation to Europe requires adopting European legislation, which can generate solid commercial constraints. Narayan et al. [16] reported that in Tanzania and Nigeria, two countries highly affected by AF contamination [17], this was not a critical factor in peanuts and corn exports since the high domestic demand for these products resulted in negligible amounts being released for exportation. The same occurs in Angola and Mozambique, particularly in rural areas, where the trade of these staples is residual and they are mainly produced for family consumption or local trade. This contributes to the domestic consumption of highly contaminated products, making these populations even more susceptible to high AF intake.

Agriculture is a fundamental activity affecting most families in both Angola and Mozambique. Family farms constitute around 98% of all farms, and 60 to 75% of the population depends on agriculture for survival [18,19]. It is estimated that there are 2.3 million and 4.2 million family farms in Angola and Mozambique, respectively [18,19]. Staple foods like corn, peanuts, rice, cassava, and beans are not only the basis of family nutrition in these countries; they are also sources of economic income, mainly resulting from local formal or informal trade. In Angola, the province of Cuanza Sul has the third-largest farming area and the third-highest number of farms in the country, and 97% of the families depend on agriculture [19]. In this province, the most significant crops are corn, produced on 95% of the farms, cassava (57%), and beans (56%), followed by peanuts (36%) [19]. In Mozambique, a significant number of farms produce corn (83.8%), peanuts (23.6%), and rice (12.8%) [18], and the south provinces of Gaza and Inhambane also depend on these staples. Gaza is the country’s third largest producer of corn and rice, while the province of Inhambane is a significant peanuts producer.

Despite the importance of agricultural production, Mozambique suffers from one of the highest malnutrition rates in the world, especially in rural areas [20]. In 2019, almost 30% of the families faced acute food insecurity; in comparison, 16% was reported in 2016 [21]. In 2019, the provinces of Gaza and Inhambane registered the highest levels of acute food insecurity in the country, with 48% and 40% of families facing this problem, numbers that can be compared against the values of 39% and 20% registered in 2016 [21]. In Angola, the level of food insecurity is also alarming, and malnutrition is a public health issue affecting almost half of the population, with around 1.58 million people suffering from acute severe food insecurity [22].

Post-harvest food losses, including those resulting from fungal growth and mycotoxin accumulation, are among the most important causes of food insecurity and malnutrition in these countries [23]. MADER [18] estimates that corn losses in the provinces of Maputo and Inhambane, Mozambique, reach as high as 29.4% and 26% of the production, respectively, thus strongly contributing to food supply shortages. Also, AF exposure among young children is correlated with impaired growth and stunting [3] and strongly correlated with hepatocellular cancer [24]. The adoption of strategies for mitigating AF contamination in food is thus fundamental for food security, public health, and commercial reasons and will aid in reducing extreme food insecurity situations.

This study aimed to analyze the total AF contamination levels of various staple foods produced by small-scale and subsistence farmers in rural settings of the province of Cuanza Sul, Angola, and the provinces of Gaza and Inhambane, South Mozambique, to understand the extent to which AFs pose a risk in these two countries. To the best of the authors’ knowledge, this is the first report on AF contamination of foods from Angola.

## 2. Results

**Figure 1:**
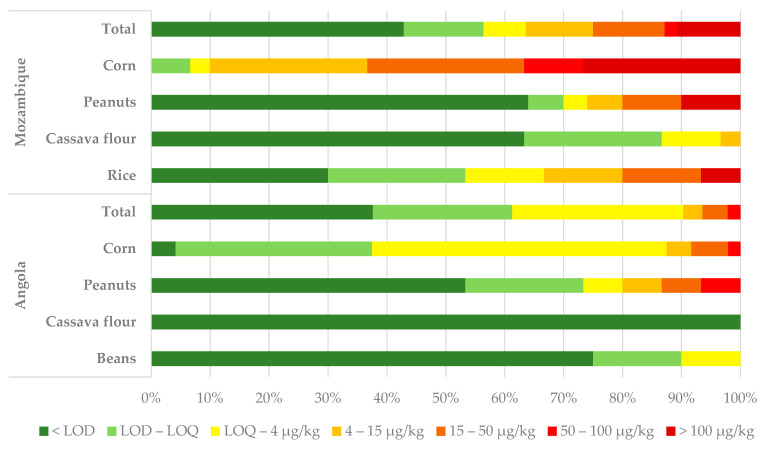
Distribution of samples (as percentages) according to level of contamination.

This work looked at the incidence of total aflatoxin (AFT) contamination in various staple foods—corn, peanuts, beans, rice and cassava flour—produced in the province of Cuanza Sul (five districts), Angola, and in the provinces of Gaza (three districts) and Inhambane (three districts), South Mozambique. The overall occurrence and average, median, and range (min and max) levels of AFT are reported in Table 1. Table 2 provides a detailed description of the incidence and levels of AFT contamination by country, province, district, and product, as well as the percentage of samples exceeding the maximum tolerable limits set by the European Union (MTL-EU) and the Codex Alimentarius (MTL-Codex). Figure 1 reports the distribution of samples (in percentages) according to class of contamination.

Overall, the results show that AFTs were detected in all the analyzed crops, except cassava from Angola, where no contamination was detected (Table 1). Disregarding the origin of the samples, corn was the crop with the highest incidence and level of contamination, followed by peanuts and rice. Beans and cassava flour were the least contaminated products.

### 2.1. Incidence of Total Aflatoxins in Angola

**Figure 2:**
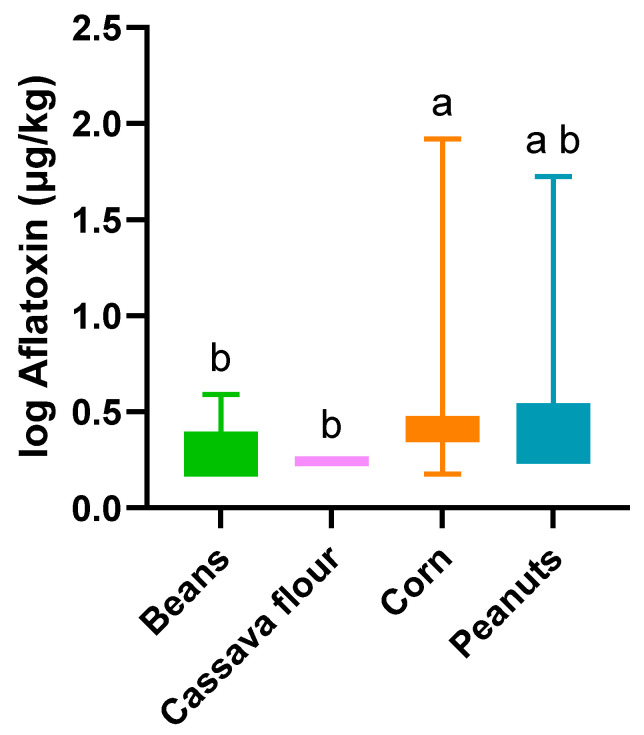
Boxplot of the average total aflatoxin concentration (1 + log10 µg/kg) in various Angolan staple foods. The bars represent the minimum and maximum intervals; different letters highlight significant differences (*p* < 0.05) between the matrices according to the Kruskal–Wallis test, followed by Dunn’s multiple comparison.

In Angola, AFT contamination was noted in all the matrices studied except cassava flour (Table 1; Figure 1), with 58 out of the total 93 samples (62%) showing detectable AFs. However, only corn and peanuts showed levels of contamination above those permitted by the European Union (MTL-EU) and the Codex (MTL-Codex) (Table 2). While corn showed a higher incidence than peanuts (96% and 47%, respectively), peanuts showed a higher average of contamination (8.3 µg/kg against 5.1 µg/kg) (Table 1; Figure 2) and a higher percentage of samples exceeding the MTLs (Table 2). The incidence of AFTs in corn produced in Angola was very high and ranged from 90% to 100% in the positive (>LOD) samples.

**Figure 3:**
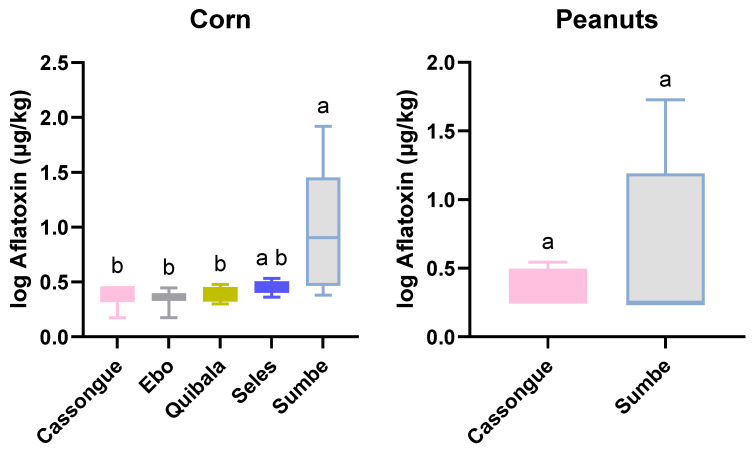
Boxplots of the average total aflatoxin concentrations (1 + log10 µg/kg) in Angola for corn (five districts, Kruskal–Wallis test, followed by Dunn’s multiple comparison) and peanuts (two districts, Mann–Whitney U test). The bars represent the minimum and maximum intervals; different letters highlight significant differences (*p* < 0.05) between the districts for corn and peanuts.

The district of Sumbe showed the highest AFT levels, with 60% and 50% of the corn samples collected there exceeding the MTL-EU (4 µg/kg) and the MTL-Codex (15 µg/kg) levels, respectively, with a maximum amount of 82.3 µg/kg (Table 2; Figure 1). AFT levels of corn in this district were significantly higher than in the remaining districts (*p* < 0.046), where the maximum levels ranged from 1.9 to 2.4 µg/kg (Figure 3) and none of the samples exceeded the MTLs. Also, in the district of Sumbe, peanuts samples exceeded the MTL-EU (4 µg/kg) and MTL-Codex (10 µg/kg) levels by around 30% and 20%, respectively, with the maximum level of AFs being 52.3 µg/kg (Table 2; Figure 3). The highest levels of contamination in the district of Sumbe may result from the samples having been collected from local markets, without knowledge of the time and conditions of storage of the products. For the remaining districts, samples were collected from the producers, either from a field or storage house. Beans and cassava flour were the least contaminated products in the province in terms of AF incidence and concentration. None of the samples reached the MTLs.

### 2.2. Incidence of Total Aflatoxins in Mozambique

**Figure 4:**
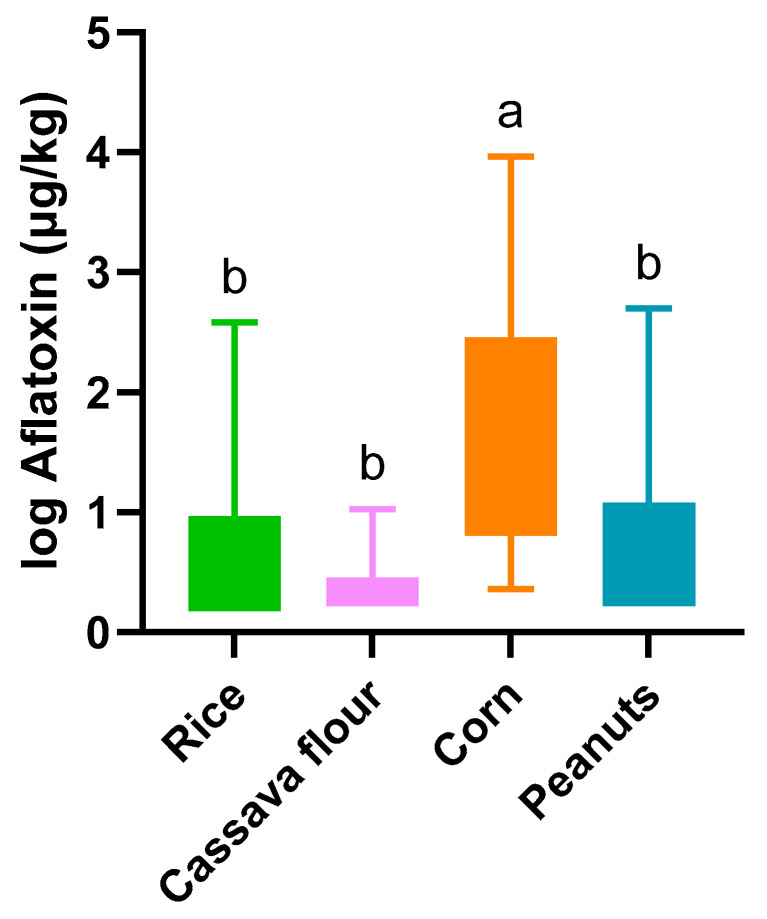
Boxplot of the average total aflatoxin concentrations (1 + log10 µg/kg) in various staple foods from Mozambique. The bars represent the minimum and maximum intervals; different letters highlight significant differences (*p* < 0.05) between the matrices after analysis via Kruskal–Wallis test, followed by Dunn’s multiple comparison.

In Mozambique, AFTs were detected in all the matrices studied. Corn and rice had the highest incidences (Table 1; Figure 1)—100% and 70%, respectively. Corn showed significantly higher contamination than the other crops analyzed (Figure 4). Cassava showed the lowest incidence and AFT levels, and no samples exceeded the MTLs (Table 2).

**Figure 5:**
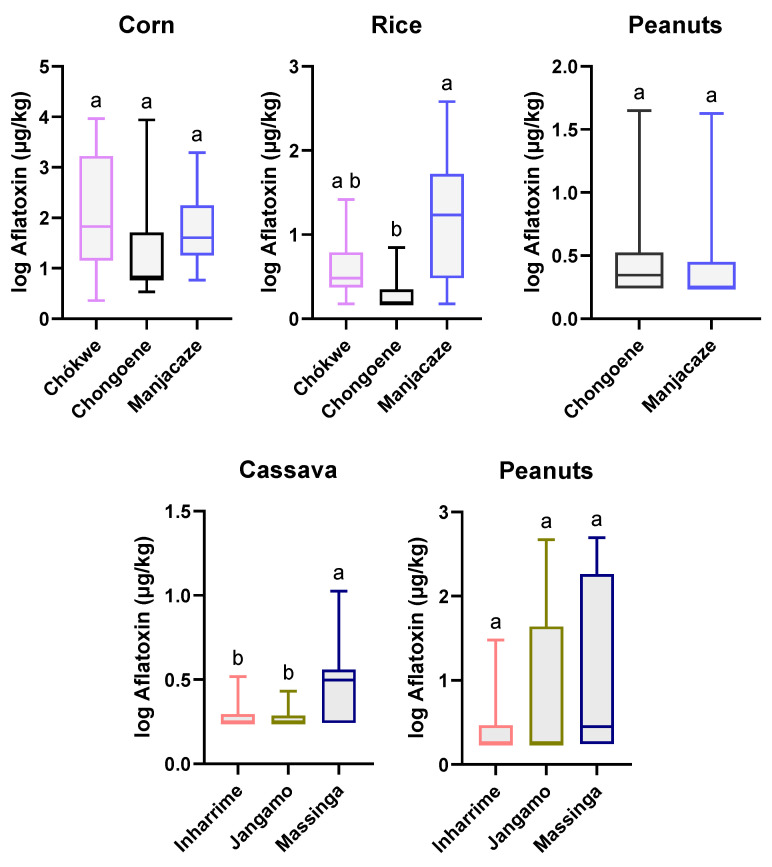
Average total aflatoxin concentrations (µg/kg) in various staple foods of Mozambique in the six districts (top: province of Gaza; bottom: province of Inhambane). Error bars represent the minimum and maximum intervals. Different letters highlight statistical differences (*p* < 0.05) between districts (determined via Kruskal–Wallis test, followed by Dunn’s multiple comparison, for all matrices except for peanuts in Gaza, where the Mann–Whitney U test was applied).

In Mozambique, rice and corn were only collected in Gaza Province since these crops are not significant in the province of Inhambane. On the other hand, cassava flour was only sampled from Inhambane. Among the sampled products, only peanuts were collected from both provinces. The incidence of Afs in corn produced in Mozambique reached 100% in the three districts of Gaza province (Table 2). Because of the high variance, the differences between districts were not statistically significant (*p* > 0.05), but Chokwe district showed the highest average (1972.6 µg/kg) and median (66.5 µg/kg), with two samples reaching AFT contamination levels as high as 9200 µg/kg (Table 2; Figure 5).

In the districts of Chokwe and Manjacaze, more than 80% of the corn samples exceeded the MTL-EU (4 µg/kg) and MTL-Codex (15 µg/kg) levels (Table 2). Concerning rice, the highest incidence was observed in the district of Manjacaze, Gaza Province, with a positive rate of 90% and 60% exceeding the MTL. Manjacaze samples were significantly (*p* < 0.05) more contaminated than those from Chongoene and Chokwe (Figure 5). Peanuts had the highest incidence (50%) and median (2.2 µg/kg) in the district of Massinga, Inhambane Province, with the highest absolute level being 496 µg/kg (Table 2). Nonetheless, there were no significant differences between districts. Cassava flour (locally named rali) was the least contaminated product sampled in the country. It had the highest incidence in the district of Massinga, Inhambane Province, with a positive rate of around 70% but only one sample exceeding the MTLs (Table 2). The level of contamination in cassava flour from Massinga was statistically higher (*p* < 0.05) than that from Jangamo and Inharrime (Figure 5).

### 2.3. Comparison of Crops Between Angola and Mozambique

**Figure 6:**
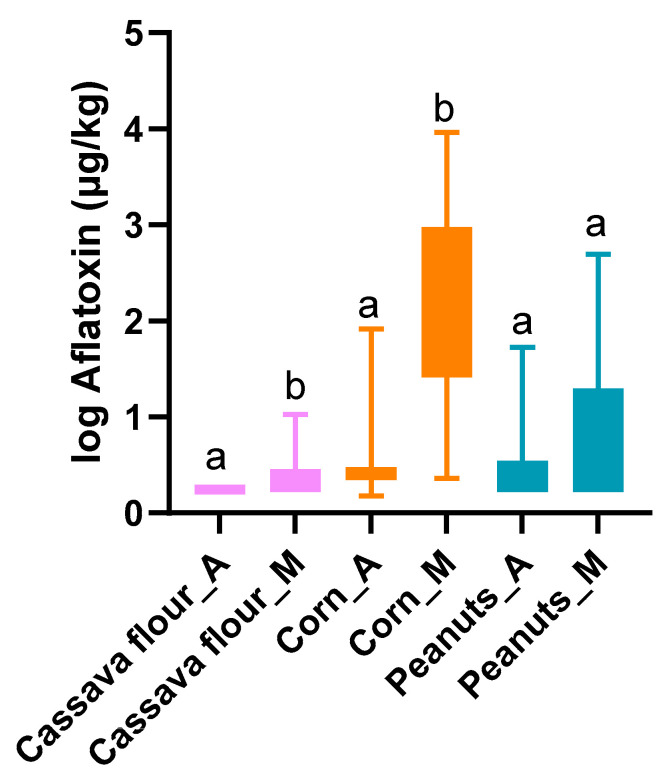
Comparative analysis of the average total aflatoxin concentrations (1 + log10 µg/kg) in common staple foods from Angola (“staple”_A) and Mozambique (“staple”_M). Error bars represent minimum and maximum values, and different letters highlight statistical differences (*p* < 0.05) between the same staple for both countries (Mann–Whitney U test).

All crops sampled from both countries—corn, peanuts, and cassava—showed higher contamination in Mozambique than in Angola (*p* < 0.0071). Corn and cassava from Mozambique stood out in terms of contamination compared to Angola (*p* < 0.001 and *p* = 0.028, respectively), while for peanuts, there were no significant differences (*p* = 0.677) (Figure 6). For corn, it is noticeable that the risk was much higher in Mozambique.

## 3. Discussion

Due to climate vulnerability, Mozambique is considered one of the countries most affected by AF [25]. However, few studies have analyzed AF contamination in Mozambican food products [7,8,9,10,11,12,13]. Furthermore, to the best of our knowledge, this is the first study on AF contamination in food staples produced in Angola.

This study found a high level of AFT contamination in corn produced in Mozambique and Angola, with AFT values from Mozambique reaching 9200 μg/kg in two samples and one reaching 8736 μg/kg, while in Angola, the highest detected value was 82.3 µg/kg. Corn is one of the world’s most important food staples, and in both Angola and Mozambique, it is the agricultural product with the highest dietary and economic relevance. Because of its importance and particular susceptibility, corn is also one of the most studied food crops for AF contamination [17]. In general, in the various products analyzed in many African countries, AFB1 contamination levels are relatively high. In a systematic review, Meijer et al. [17] found that among the 27 analyzed studies, 25 indicated a mean AFB1 level in corn of > 5 μg/kg. El-Shanshoury et al. [26] reported a mean AFB1 concentration of 440 μg/kg in corn from Egypt, and individual samples with values as high as 6738 μg/kg (AFB1) (from Nigeria) [27], 9091.8 µg/kg (AFB1) (from Kenya) [28], 3760 µg/kg (AFT) (from Uganda) [29], and up to 2806.5 µg/kg (AFT) (from the Democratic Republic of Congo) [30] have been reported. Few studies have reported low mean AF concentrations. Martinho et al. [10] investigated 30 samples of corn flour collected at milling factories in Nampula, Mozambique, and detected AFT in very low concentrations: an average of 0.89 µg/kg, and a maximum level of 1.05 µg/kg.

Climate remains one of the main driving factors of mycotoxin production in foods from Sub-Saharan Africa [31]. Nonetheless, post-harvest factors strongly influence AF accumulation in corn due to the characteristics of the AF-producing fungi. Corn produced by subsistence farmers is considered to be at a higher risk of contamination due to particularly poor drying and storage conditions [32]. In Angola and Mozambique, the common post-harvest drying and storage methods include no or residual sorting of moldy grains, long drying periods at high temperatures and under high humidity, and unhygienic storage conditions, with access of rodents and insects to the storage facilities. It is recommended that corn should be under conditions with less than 15% humidity within 10 days of harvesting to avoid contamination by aflatoxins [33]. Given the climate conditions, this is not always achieved, and many more days of high humidity and rain can pass until the corn is properly dried, and it is often stored before being properly dried. Hermetic technologies are recommended for storage [34], but this technology is seldom available to subsistence farmers. While it is difficult for these farmers to address climate conditions, drying and storage can be adjusted if farmers are properly supported with materials and training [31].

The incidence and levels of AFT detected in this study in peanuts from Angola and Mozambique were also high but significantly lower than those for corn, and few samples exceeded the international MTLs. The maximum values found were 496 μg/kg in one sample from Mozambique and 52.3 μg/kg in one sample from Angola. Previous studies have reported contamination of Mozambican peanuts with AFT. A study of 23 samples from local markets in the province of Nampula, in the north of the country, found median levels of AFT of 3.4 μg/kg, within the range of 3.4−123 μg/kg [8]. In 2018, 57 market and supermarket samples of raw peanuts from Maputo, the capital of Mozambique, were found to have AFB1, with average values of 2.71 µg/kg and a maximum of 72.93 µg/kg [35]. Bila et al. [12] tested 47 samples of peanuts from the provinces of Gaza and Inhambane and found AFT contamination in 83%, with averages ranging from 1.43 to 10.85 µg/kg and a maximum value of 17.42 µg/kg. In a neighboring country, Tanzania, AF contamination in peanuts is also widespread, and 96.1% of the 180 samples analyzed by Boni et al. [36] were contaminated, with AFT values up to 10.93 µg/kg. In the Democratic Republic of Congo, the occurrence and levels of AF, mainly AFB1, in raw peanuts were significantly higher, with values up to 937 µg/kg, and tended to increase from the dry season to the rainy season [37]. The lack of detailed scientific knowledge on the extent of the AF problem in peanuts and the associated health risks is still a challenge for Mozambique and Angola. Ours is not an isolated case; in many countries, there are still gaps in knowledge or evidence regarding AF in peanuts and peanuts products, and, therefore, there is a need to promote more research to fill these gaps [38].

Considering cassava flour, our samples’ contamination levels were low or even undetectable. While no samples from Angola showed contamination, 11 out of 30 samples (37%) from Mozambique showed AFT contamination with average values of 2.8 μg/kg, and only one exceeded the MTL-EU. Fresh cassava is not usually associated with AF contamination, even when aflatoxigenic fungi are present, due to anti-aflatoxigenic compounds [37], but inadequate drying, processing, and storage conditions might result in the loss of these properties and favor the development of aflatoxigenic fungi [39,40]. Many studies from Nigeria, Malawi, Zambia, Benin, Uganda, and Tanzania have shown little or no AF contamination in processed cassava products [39,41,42,43,44,45,46], while others from the Republic of Congo and Benin found incidences as high as 100%, with values up to 9 μg/kg [47,48]. In Cameroon, Essono et al. [49] evaluated 72 samples of cassava chips over two months of storage. Of the total, 18 samples showed AF contamination with a variation between 5.2 and 14.5 μg/kg, but only after four weeks of storage. In Tanzania and the Republic of Congo, Manjula et al. [48] reported AFB1 contamination levels from 0.3 to 4.4 μg/kg in cassava chips and flour, and from 0.1 to 13.0 μg/kg in stored cassava samples, with relatively high levels of contamination found in cassava stored for 4 months.

The rice produced and consumed in Mozambique (mainly in Manjacaze) is contaminated with AFT at levels that exceed the limits tolerated by European Union legislation and the Codex Alimentarius. Although high variability in AF contamination in rice has been observed worldwide, the highest contamination levels have been reported in developing countries, mainly those in Asia [50]. Few studies report on the presence of AFs in rice produced in Sub-Saharan Africa. In Nigeria, two studies reported that 100% of the analyzed rice samples were deemed unsafe (all exceeded the MTLs), with AF values within the ranges of 37–112 μg/kg [51] and 28–372 μg/kg [52].

Aflatoxin contamination in beans in Angola was not significant in this study and did not exceed the limits set by the European Union or Codex Alimentarius, although there was an around 40% positivity rate. Beans have not been vigorously studied in terms of AF contamination, and contradictory results have been reported. A study from Nigeria reported a 58% incidence of AF in beans, with mean values as high as 63–106 μg/kg [53]. On the contrary, another study from Nigeria reported that among 15 samples of bean flour sold commercially, 9 were contaminated with AFs but at residual levels below 0.151 μg/kg [54].

Our results show a high level of AFT in Mozambique and Angola’s main matrices considered staple foods. While cassava and beans seem less affected by AF contamination, there is an urgent need to adopt mitigation strategies to minimize AF contamination in corn, peanuts, and rice because these are fundamental staples in Africa in terms of its food and agricultural economy.

Aflatoxins affect several countries in the world and the SADC region, and their high incidence in southern African countries constitutes a considerable concern for food security in the region. The SADC’s vulnerability to climate change is not caused by climate change alone; it is a combination of social, economic, and other environmental factors that interact with climate change [31,32,55]. The situation in Angola and Mozambique is like that in other countries with a tropical climate and that are just as vulnerable to climate change [56]. The hot and humid climates in tropical and subtropical regions are favorable for the growth of aflatoxigenic fungi, and these conditions lead to the prevalence of AF in many agricultural products [4,57].

Considering the maximum admissible levels for AFT recommended by the European Commission and the Codex Alimentarius Commission for cereals and pulses, the levels of AFT contamination in staple foods produced and consumed in Angola (Cuanza Sul) and Mozambique (southern) are high and constitute a public health risk for the population. Thus, urgent mitigation measures are required to guarantee food safety for the population.

## 4. Materials and Methods

### 4.1. Study Site

**Figure 7:**
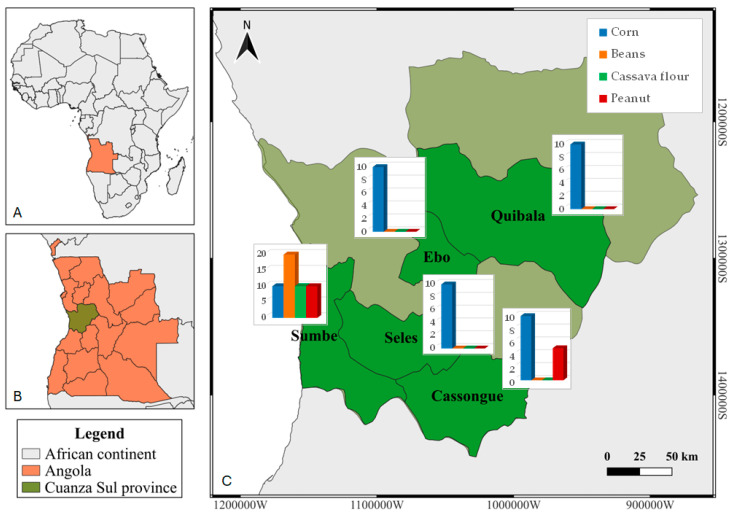
Map of Angola showing the sample collection sites: (**A**) African continent, with Angola highlighted in orange; (**B**) Angola, with the province Cuanza Sul highlighted in green; and (**C**) Sampled districts, highlighted in dark green, with the number of samples of each sampled agricultural product.

**Figure 8:**
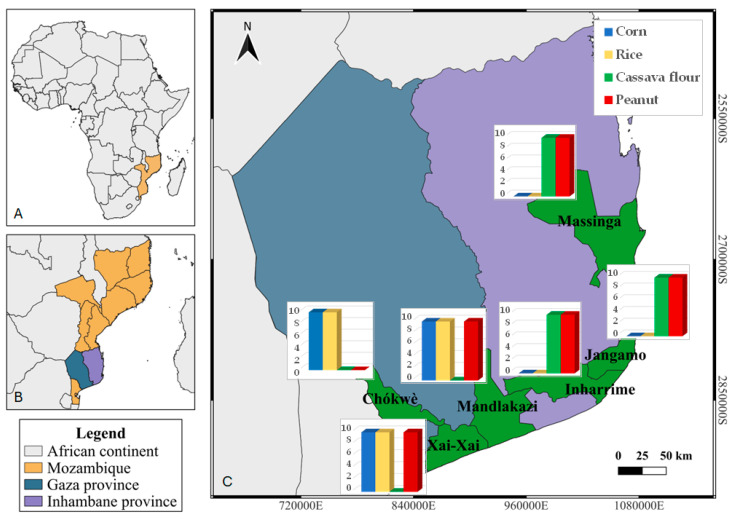
Map of Mozambique showing the sample collection sites: (**A**) African continent, with Mozambique highlighted in orange; (**B**) Mozambique, with the province of Gaza highlighted in blue and the province of Inhambane highlighted in purple; and (**C**) sampled districts, highlighted in green, with the number of samples of each sampled agricultural product.

Samples of locally produced staples were collected from local markets in Angola and in Mozambique in June–August 2022. In Angola, samples were collected in five districts of the province of Cuanza Sul—Cassongue, Quibala, Ebo, Seles, and Sumbe (Figure 7). In Mozambique, samples were collected from three districts of the province of Gaza—Chókwè, Manjacaze (also known as Mandlakazi), and Chongoene (previously the district of Xai-Xai)—and from three districts of the province of Inhambane—Jangamo, Inharrime, and Massinga (Figure 8).

### 4.2. Sampling

The most significant staples from each region were selected for sample collection based on consumption questionnaires administered to families and production questionnaires administered to farmers of the sampled districts/municipalities (data not published) and from published reports from both countries, namely, the Integrated Agrarian Report 2020 [18] from Mozambique and the Agro-Livestock and Fisheries Census 2022 (Recenseamento Agro-Pecuário e Pescas [19]) from Angola. Based on these supporting reports, corn, peanuts, and rice were selected as the most significant staples from the studied provinces of Mozambique with different regional distributions. As reported by MADER [18] for the year 2020, the most significant cereals produced in the province of Gaza were corn (64,763 t) and rice (17,757 t), while Inhambane produced mainly corn (15,885 t). No rice production was reported in Inhambane in 2020. Peanuts were also an important staple in both provinces (4773 t in Gaza and 5493 t in Inhambane). Even though the two selected provinces are not the major staple producers in this country, they are of great interest for this study since (i) they are mostly represented by small to medium-sized farms; (ii) farmers here show the lowest school education and agrarian training levels in the country; (iii) most of the produced staples are for family consumption, since only small portions of the produced goods are sold, namely, 1.2% (Gaza) to 7.0% (Inhambane) of corn (country average: 18%), 10% (Gaza) of the rice (country average 16%), and 0.2% (Gaza) to 0.3% (Inhambane) of the peanuts (country average 20%); (iv) post-harvest losses are among the highest in the country for corn—13.8% in Gaza and 26% in Inhambane (country average 13.5%) and 29% for peanuts in both provinces (country average: 24.5%) [18]. Among the various staple foods in Angola, corn, cassava, beans, and peanuts were selected according to their importance in the country. Corn is the most important product in Cuanza Sul and is grown by 95% of farmers, while cassava is grown by 57%, butter beans are grown by 56%, and peanuts are grown by 36% [19].

A total of 233 samples were collected from the two countries (140 from Mozambique and 93 from Angola), as described in Figure 7 and Figure 8. Approximately 1 kg of each sample was bought from the sellers (via local markets) or producers (via fields or storage houses) and transported in paper bags to the Microbiology Laboratory of Instituto Superior Politécnico de Cuanza Sul, in the case of Angola, and the Xai-Xai Water and Food Laboratory, in the case of Mozambique. A representative 200 g subsample was conditioned in paper bags and transported to the Mycology Laboratory of the Centro de Investigação de Montanha, Bragança, Portugal, for analysis.

### 4.3. Aflatoxins Analysis

One hundred grams of each sample was homogenized and ground to a fine flour using a Vevor grinder (model XZ-68, Shanghai, China). Total aflatoxins were analyzed using the lateral flow AgraStrip Pro WATEX^®^ (Romer Labs, Tulln, Austria) validated procedures for each matrix, as described by the manufacturer [58].

Briefly, 10 g ± 0.1 g of ground sample, one buffer bag, and 50 mL of deionized water were added to a Whirl-Pak^®^ filter bag (1:5 (*w*/*v*) extraction ratio). The mixture was shaken vigorously for 2 min and then allowed to settle for 1 min. The supernatant (100 µL) was transferred into a microcentrifuge tube and mixed with the appropriate volume of dilution buffer, depending on the matrix (as set in the validation procedure for each matrix). The diluted sample was centrifuged at 2000× *g* for 30 s, and 200 μL of the sample extract was pipetted into the cartridge. All buffers, bags, microcentrifuge tubes, pipette tips, and cartridges were provided in the kit.

The limit of detection (LOD), the limit of quantification (LOQ), and the upper detection limit (UDL) are shown in Table 3. Quantification was performed using the AgraVision™ Pro Reader (Romer Labs, Tulln, Austria). Whenever necessary, sample extracts were diluted with the kit’s diluent, and the analysis was repeated.

### 4.4. Statistical Analysis

For the quantitative analysis of the data (conducted to calculate the average and the median), since the LOD and LOQ were available, for results lower than the LOD, the value LOD/2 was used, and for those between the LOD and the LOQ, the obtained LOQ value was used, as recommended by [59].

AFT values were log-transformed [y = log10 (1 + AFT), µg/kg] to normalize variances. The variances of the means of the mycotoxins among countries, districts, and crops were compared using non-parametric Kruskal–Wallis one-way ANOVA (95% confidence interval), followed by Dunn’s multiple comparisons test (for 3 or more comparisons) or the Mann–Whitney U test (for 2 comparisons), using GraphPad Prism version 10.4. The Kruskal–Wallis test was used because the data did not meet the assumptions of normality and homogeneity required by the Analysis of Variance F-test. StatSoft Inc. STATISTICA version 12 www.statsoft.com (accessed on 15 September 2024) was used for statistical analyses.

## Figures and Tables

**Table 1:** Overall results for total aflatoxin contamination of samples from Angola and Mozambique, arranged by product.

	N	Positives (%)	Average of All Samples (µg/kg)	Median (µg/kg)	Range (µg/kg)
Corn					
Mozambique	30	100	1107.6	26.9	<LOQ—9200
Angola	48	96	5.1	1.7	<LOD—82.3
Total	78	97	429.1	2.0	<LOD—9200
Peanuts					
Mozambique	50	36	40.6	<LOD	<LOD—496
Angola	15	47	8.3	<LOD	<LOD—52.3
Total	65	39	33.2	<LOD	<LOD—496
Cassava					
Mozambique	30	37	1.5	<LOD	<LOD—9.6
Angola	10	0	<LOD	-	<LOD
Total	40	28	1.3	<LOD	<LOD—9.6
Beans					
Mozambique	-	-	-	-	-
Angola	20	25	<LOD	<LOD	<LOD—2.9
Total	20	40	<LOD	<LOD	<LOD—2.9
Rice					
Mozambique	30	70	23.1	1.8	<LOD—380
Angola	-	-	-	-	-
Total	30	70	23.1	1.8	<LOD—380
All samples					
Mozambique	140	57	257.1	1.7	<LOD—9200
Angola	93	62	4.3	1.3	<LOD—82.3
Total	233	59	156.2	1.5	<LOD—9200

**Table 2:** Detailed description of the incidence and levels of total aflatoxin contamination in Angola and Mozambique according to country, province, district, and product. Colors represent the level of contamination, ranging from lowest (green) to highest (red).

Country	Province	District	Product	Total Samples (n)	Positive Samples (n)	Positive Samples (%)	Average of All Samples (µg/kg)	Average of Positive Samples (µg/kg)	Median of All Samples (µg/kg)	Median of Positives Samples (µg/kg)	Min (µg/kg)	Max (µg/kg)	Samples > MTL Codex (%)	Samples > MTL EU (%)
Angola	Cuanza Sul	Cassongue	Corn	10	9	90	1.5	1.6	1.7	1.7	1.0	1.9	0.0	0.0
			Peanuts	5	3	60	1.3	1.3	1.2	1.2	1.0	1.8	0.0	0.0
		Ebo	Corn	10	9	90	1.5	1.5	1.5	1.5	1.0	2.0	0.0	0.0
		Quibala	Corn	10	10	100	1.5	1.5	1.5	1.5	1.0	2.0	0.0	0.0
		Seles	Corn	8	8	100	1.9	1.9	2.0	2.0	1.3	2.4	0.0	0.0
		Sumbe	Corn	10	10	100	18.6	18.6	7.7	7.7	1.4	82.3	50.0	60.0
			Beans	20	5	25	1.0	2.3	0.5	1.9	1.6	2.9	0.0	0.0
			Cassava flour	10	0	0	<LOD	<LOD	<LOD	<LOD	<LOD	<LOD	0.0	0.0
			Peanuts	10	4	40	11.7	28.2	0.8	29.2	2.2	52.3	20.0	30.0
														
Mozambique	Gaza	Chokwe	Corn	10	10	100	1972.6	1972.6	66.5	66.5	1.3	9200.0	80.0	80.0
			Rice	10	9	90	4.9	5.3	2.1	2.2	1.0	25.2	10.0	10.0
		Manjacaze	Corn	10	10	100	369.2	369.2	40.0	40.0	4.8	1950.0	90.0	100.0
			Peanuts	10	2	20	5.8	26.0	0.8	26.0	10.6	41.3	20.0	20.0
			Rice	10	9	90	63.1	70.0	17.3	23.7	1.4	380.0	60.0	60.0
		Chongoene	Corn	10	10	100	981.1	981.1	5.8	5.8	2.4	8736.0	30.0	90.0
			Peanuts	10	5	50	5.6	10.5	1.3	2.3	1.8	43.6	10.0	10.0
			Rice	10	3	30	1.2	2.9	0.5	1.7	1.1	6.0	10.0	10.0
	Inhambane	Jangamo	Peanuts	10	4	40	95.5	237.6	0.8	235.5	12.6	467.0	40.0	40.0
			Cassava flour	10	2	20	0.9	1.7	0.8	1.7	1.6	1.7	0.0	0.0
		Inharrime	Peanuts	10	2	20	4.8	20.9	0.8	20.9	12.6	29.1	20.0	20.0
			Cassava flour	10	2	20	1.0	2.1	0.8	2.1	1.8	2.3	0.0	0.0
		Massinga	Peanuts	10	5	50	91.4	182.1	2.1	173.0	3.5	496.0	40.0	40.0
			Cassava flour	10	7	70	2.6	3.3	2.2	2.5	1.7	9.6	0.0	10.0

**Table 3:** Limit of detection (LOD), limit of quantification (LOQ), and upper detection limit (UDL) (in µg/kg) for the method used for total aflatoxin analysis.

Matrix	LOD	LOQ	UDL
Beans	1.0	1.5	50
Cassava flour	1.5	2.6	10
Corn	1.0	1.5	50 or 100
Peanuts	1.5	2.5	50
Rice	1.0	2.0	50

## Data Availability

The original contributions presented in this study are included in the article. Further inquiries can be directed to the corresponding author(s).
